# Associations between poor oral health and reinjuries in male elite soccer players: a cross-sectional self-report study

**DOI:** 10.1186/s13102-015-0004-y

**Published:** 2015-04-20

**Authors:** Henny Solleveld, Arnold Goedhart, Luc Vanden Bossche

**Affiliations:** 1 SportsInjuryLab, Box 3141, 3760 DC Soest, The Netherlands; 2 Physical Rehabilitation and Sports Medicine, Ghent University Hospital, De Pintelaan 185, 9000 Ghent, Belgium

**Keywords:** Sports injuries, Soccer, Oral health, Gingival diseases, Dental caries, Dental plaque, Psychosocial factors

## Abstract

**Background:**

Although it is well known that oral pathogens can enter the systemic circulation and cause disease, it is largely unknown if poor oral health increases the risk of sports injuries. The purpose of this study is to investigate the association between poor oral health and reinjuries in male elite soccer players, adjusted for psychosocial problems and player characteristics.

**Methods:**

184 Players in premier league soccer clubs and 31 elite, junior soccer players in the Netherlands, Belgium and England, were enrolled in a retrospective cross-sectional study. The Sports Injury Risk Indicator, a self assessed questionnaire, was used to obtain information on reinjuries, age and player position, oral health and psychosocial problems. The number of different types of oral health problems was used as an indicator of poor oral health. (SumDental, range 0–2: 0 = no oral health problems, 1 = one type of oral health problem and 2 = two or more types of oral health problems). Multivariable logistic regression was used to investigate whether SumDental was associated with reinjuries, after adjustment for psychosocial problems and player characteristics.

**Results:**

37% of the players reported no oral health problems, 43% reported one type of oral health problem and 20% reported two or more types of oral health problems. After full adjustment for age, player position and psychosocial problems (i.e. injury anxiety, psychophysical stress, unhealthy eating habits and dissatisfaction with trainer/team), poor oral health (SumDental) was positively associated with all kind of reinjuries whether analyzed as a continuous variable or as a categorical variable. The fully adjusted odds ratios for SumDental analyzed as a continuous variable were: in relation to repeated exercise-associated muscle cramps: 1.82 (95% confidence interval (CI): 1.07, 3.12), in relation to muscle or tendon reinjury 1.57 (95% CI: 1.01, 2.45) and in relation to multiple types of reinjury 1.88 (95% CI: 1.19, 2.97).

**Conclusion:**

The results from this study justify a thorough examination of the effects of oral health problems on the injury risk of playing elite soccer.

## Background

Injuries in male elite soccer players are common, with muscle and tendon injuries comprising almost half of all injuries [1]. On average, one injury occurs every 106 hours of sport-related activity and 65 - 95% of players have at least one injury every year [1-3]. The injury model of Bahr and Krosshaug states that intrinsic and extrinsic risk factors and the interaction between them make players susceptible to an injury caused by events such as jumping, tackling and shooting [4]. The intrinsic risk factors proposed by Bahr and Krosshaug comprise health, including previous injury, age, physical fitness and psychological factors. Poor oral health is not included as an intrinsic risk factor in the injury causation model. This is noteworthy because it is widely accepted that good oral health is essential for good general health [5]. Many studies have shown that dental diseases are associated with systemic illness including cardiovascular diseases (CVDs) and various other pathologies [6]. The well-known MilanLab of AC Milan includes an assessment of oral health in the injury prevention program. However, we are aware of only one study that has investigated the possible impact of dental and occlusal problems on sports injuries. Gay-Escoda et al. found in their study of 30 professional soccer players of F.C. Barcelona, that the Plaque Index and periodontal pocket depth were associated with muscle injuries [7].

The most prevalent oral diseases in humans, periodontitis and dental caries, are dental plaque related diseases [8]. Dental plaque is a microbial biofilm formed by microorganisms tightly bound to a tooth surface and each other. The microbial composition is diverse and remains relatively stable over time. However, when this microbial homeostasis breaks down oral disease can occur [8,9]. Oral disease cause elevated levels of cytokines, especially tumour necrosis factor (TNF-a) and interleukin-6 (IL-6). These cytokines play an important role in the origin of muscle fatigue during exercise and oxidative stress after exercise [10-13]. Muscle fatigue may cause exercise-associated muscle cramps and leads to a reduction in its energy-absorbing capabilities, making the muscle more susceptible to strain injury [14,15]. In addition, muscle fatigue increases the possibility of proprioceptive errors and disturbances in the interactions between limb segments [16,17]. Therefore, oral disease is a potential risk factor for sports injury and reinjuries.

Risk factors associated with oral disease include poor oral hygiene, cigarette smoking, systemic conditions such as diabetes mellitus, rheumatoid arthritis, possibly obesity, stress and poor coping behaviours [18,19]. Stress and poor coping behaviours are also risk factors for sports injuries. Many studies have found evidence for the stress-injury model of Williams and Andersen [20]. This model suggests that stress is associated with a increased risk of injury and is directly, as well as indirectly, influenced by personality traits and coping resources. As these psychosocial factors are related to both sports injuries and poor oral health, associations between poor oral health and sports (re)injuries are compounded by these factors.

Stress has been etiologically linked to occlusal and temporomandibular joint (TMJ) problems [21,22]. Individuals with high levels of stress display muscular tension and parafunctional habits like bruxism that give rise to jaw joint problems. Occlusal and TMJ problems may therefore be regarded as physical signs of stress.

This study focuses on reinjury because of the stability of oral conditions [8,9] and because previous sports reinjuries will probably be more accurately reported than previous sports injuries. The aim of this retrospective cross-sectional study is (1) to assess the association of poor oral health with sports reinjuries and repeated exercise-associated muscle cramps (REAMC) in male elite soccer players; and (2) to determine whether the association of poor oral health with sports reinjuries and REAMC persisted after adjustment for other possible risk factors.

## Methods

### Procedure

The study was conducted between September 2011 and December 2012. Five Dutch clubs, four Belgian clubs and one British premier league club were invited to participate in this study using medical staff known to two of the researchers (HS and LvdB) and via acquaintances of these acquaintances. The clubs were asked to present a questionnaire to all players from the first team squads (there were no eligibility criteria). In addition, one Belgian and one Dutch club were also asked to present the questionnaire to their elite junior squads. Two Dutch clubs refused to participate with their elite squad. The average number of players of first team squads is about 30 for Belgian and Dutch elite soccer clubs and about 40 for British elite soccer clubs. The Belgian and Dutch elite junior squads have about 20 players each. This makes a total of about 290 soccer players potentially in the study.

The participating clubs were visited by the first author to explain the aims and procedures of the project, to gather information on the number of players and the language they spoke and to make arrangements with regard to the presentation and administration of the questionnaires. Anonymous questionnaires were presented to the players by a staff member of each participating club as a survey on a diversity of health aspects. The staff member made it clear that everyone was free to participate and to stop answering the questions at any time.

The self-report questionnaire consisted of 71 questions on reinjuries, age and player position, oral health and psychosocial problems. To prevent low response rates typically associated with questionnaires, we kept it short, with simple, straightforward questions, of which most were answered on a 4-point Likert scale. The questionnaire was tested with 12 amateur soccer players to check understanding and response time (below 30 minutes). The Dutch questionnaire was professionally translated into 9 languages (English, French, Spanish, German, Italian, Danish, Portuguese, Russian and Estonian) and then translated back into Dutch to ensure accuracy.

Ethical approval was not sought for this study which used an anonymous questionnaire. In general, ethical protection is required for research on human participants which includes identifiable human material or identifiable data [23]. In the Netherlands, research requires ethical approval only if respondents are “subjected to procedures” or required to follow “rules of behaviour”, [24]. The Belgian Advisory Committee on Bioethics states that ethical approval is not required if the conditions of “experiment” are not met, e.g. if the researchers have had no direct influence on the observed situation [25]. We concluded after a detailed review of the guidance given by the National Research Ethics Service: “Does my project require review by a Research Ethics Committee?”, that ethical approval was not required in the United Kingdom [26]. The players could read the anonymous questionnaire before starting to respond and were free to stop responding at any time. Completion of the questionnaire constituted consent to participate in the study.

### Assessment of reinjuries

The questionnaire item: “Which of the following types of injury have you had more than once (more responses possible)?” was used to assess the dependent variables *repeated exercise-associated muscle cramps (REAMC), muscle or tendon reinjury (MTR) and multiple types of reinjury (MR).* Respondents could mark ‘none’ or mark one or more of the following 11 types of reinjuries: groin, hamstring, quadriceps, Achilles tendon, muscle cramps, other muscle injuries, fracture, knee, ankle, sprain/ligament or other reinjury. The reinjury variable REAMC was scored 1 (present) if the reinjury type muscle cramps was checked and 0 otherwise. MTR was scored 1 (present) if one or more of the following types of reinjury was indicated: groin, hamstring, quadriceps, Achilles tendon and other muscle reinjuries, else MTR was scored 0 (absent). MR was scored 1 (present) if two or more types of reinjury were checked and 0 otherwise. It may be noted that in this study repeated exercise-associated muscle cramps were not included in the category muscle or tendon reinjuries.

### Assessment of oral health

Three types of oral health problems: *Gum problems, Restorations* and *Apex resections* were assessed using questionnaire items and scored as 1 = present or 0 = absent.

*Gum problems* was scored present if the respondent answered either “always” or “often” on the question “Have you gum problems (bleeding, swelling or recession)” or “no” on the question “Are your wisdom teeth removed?” (presence of wisdom teeth is a well known risk factor for periodontitis in adults because these teeth are in a area where periodontal pathogens accumulate due to the difficulty of keeping this relatively inaccessible area clean and to the qualitatively poorer soft tissue) [27].

*Restorations* was deemed present if participant answered “yes” on the question “Have you had one or more restorations (crowns and bridges included)”. We used a question about restorations instead of a question about caries as questions on utilization of dental care have been found valid in previous research [28].

*Apex resections* were scored present if participant reported one or more apex resections or endodontic microsurgeries. We used these questions instead of questions on endodontic disease because questions on endodontic therapy have been found valid in previous research [29]. A three-level ordered categorical variable, SumDental indicating poor oral health, was created by summing the three types of oral health problems. SumDental was coded as 0 if no oral health problems were present, as 1 if one type of oral health problems was present, and as 2 if two or three types of oral health problems were present.

### Assessment of other covariates

#### Injury anxiety

The injury anxiety scale assess worries and concerns about injuries resulting from soccer training activities. The scale consists of five items derived from the Tampa Scale for Kinesiophobia [30], e.g. “I am afraid that an injury will occur during training”, “Pain lets me know when to stop the training so that I don’t injure myself.”. Each item was answered on a 4-point Likert scale (1–4). The scale score was obtained by calculating the mean across the five (equal weighted) items, with higher scores indicating higher levels of injury anxiety. Cronbach’s alpha = 0.54.

#### Psychophysical stress

A measure of psychophysical stress was used to assess psychological and physical manifestations of psychological stress related to competitive sports, e.g. fear of performance failure. Items of this scale were drawn from the Sports Anxiety Scale (e.g. “I’m concerned about choking under pressure”, “I’m concerned about performing poorly”) and the Langner index (e.g. “How often are you bothered by your heart beating fast?”, “How often are you troubled with headaches or pains in the head?” [31,32] and three items asked about malocclusion and TMJ problems, i.e. “Have you lockjaw or popping?”, “Have you toothache of unknown origin?” and “Are you grinding your teeth?”. Each item was answered on a 4-point Likert scale (1–4). The scale score was obtained by averaging the responses of the twelve (equal weighted) scale items, and higher scores corresponded to higher levels of psychophysical stress. Cronbach’s alpha = 0.61.

#### Dissatisfaction with trainer/team

This scale measures the level of acceptance and satisfaction with the role in the team and the level of perceived support from the coach. The items were taken from the Athlete Satisfaction Questionnaire (ASQ) [33], e.g. “I am satisfied with my social status on the team”; “I am satisfied with the extent to which the trainer is behind me”. Each item was answered on a 4-point Likert scale (1–4); the positively worded items were reverse scored (e.g. the response ‘always’ as 1, and ‘never’ as 4) so that higher scores indicate dissatisfaction. The scale score was obtained by by calculating the mean across the six (equal weighted) items; Cronbach’s alpha = 0.78.

#### Unhealthy eating habits

This scale consists of two items, “How often do you eat unhealthy food?” and “How often are you looking for products with sugar”, scored from 1, ‘never’ to 4, ‘always’. The scale score was obtained by by calculating the mean across the two (equal weighted) items, with higher scores indicating unhealthy eating habits.

*Player Position* is a dichotomized variable with the positions external defenders (full backs), external midfielders, attacking midfielders and forwards scored as 1 and the remaining positions scored as 0, based on findings of previous studies that players on the positions scored as 1 performed significantly more sprints or covered more sprint distance than the other players [34,35]. Of the respondents, 88 (41%) played on less high-intensity positions (scored 0), 127 (59%) played on high-intensity positions (scored 1).

*Age* was categorized into five age groups: 16–20, 21–23, 24–26, 27–30 and ≥31.

### Data analysis

Frequencies and percentages were used to summarize categorical study variables; mean item scores and standard deviations were computed for the scales. Data distributions were examined for normality with the Lilliefors modification of the Kolmogorov–Smirnov test. Significant non-normality was found for all continuous covariates and SumDental. Therefore, Spearman rank correlations were used to examine relations between the covariates injury anxiety, psychophysical stress, unhealthy eating habits, dissatisfaction with trainer/team, age and SumDental. Point biserial correlations were used to examine relations between the dichotomous covariate player position and the other covariates.

Univariate logistic regression was used to assess the associations of the reinjury variables (i.e. REAMC, MTR, MR) with SumDental and with the other covariates. In univariate and multivariable logistic regression analyses the continuous covariates were analyzed as categorical variables, after division into quartiles, to avoid assumptions of linearity in the log odds and to minimize the effect of outlying values. The first quartile served as reference category in the logistic regression analyses, the other quartiles were entered as dummy variables. We analyzed SumDental as continuous variable and as categorical variable with the lowest score (0) as reference category and the other scores (1 and 2) entered as dummy variables. Multivariable logistic regression analysis was used to estimate the adjusted odds ratios for the association of SumDental with the reinjury variables, controlling for the effects of the other covariates. P-values less than 0.05 were considered statistically significant. Analyses were performed using SPSS Version 18.0 for Windows.

## Results

Out of 290 potential participants, 232 soccer players were given questionnaires and asked to fully complete it. Seventeen questionnaires were discarded because the respondent did not answer all items, leaving a final sample of 215 elite soccer players, which represents a response of about 75%.

The frequencies and percentages of ordered categorical study variables are presented in Table 1, the mean item scores and standard deviation of the scales in Table 2. As shown in Table 1, gum problems and apex resections were reported by a small minority (16% and 13%, respectively), most respondents (56%) reported one or more restorations.

**Table 1:** **Characteristics of the participants; categorical variables (n = 215)**

Characteristic	n	%
Age, years		
16 - 20 y	51	24
21 -23 y	69	32
24 - 26 y	37	17
27 - 30 y	33	15
31+ y	25	12
Playing position: High intensity position^1^	127	59
Gum problems present	34	16
Restorations present	121	56
Apex resections present	27	13
SumDental		
no type of oral health problems present (score 0)	80	37
one type of oral health problems present (score 1)	93	43
two or more types of oral health problems present (score 2)	42	20
Repeated exercise-associated muscle cramps (REAMC) present	43	20
Muscle or tendon reinjury (MTR) present	97	45
Multiple types of reinjury (MR) present	99	46

Legend: ^1^High intensity positions are: external defender (n = 34), central) attacking midfielder (n = 40), external midfielder (n = 29) and forward (n = 24); Lower intensity positions are: goalkeeper (n = 26), central defender (n = 34) and central defending midfielder (n = 26).

**Table 2:** **Characteristics of the participants; means, standard deviations and observed ranges of scale scores (n = 215)**

Scale	Mean	Standard deviation	Observed range
Injury anxiety	2.0	0.4	1.00 - 3.40
Psychophysical stress	1.4	0.2	1.00 - 2.25
Unhealthy eating habits	2.4	0.7	1.00 - 4.00
Dissatisfaction with trainer/team	1.7	0.5	1.00 - 3.83

Note: Scale scores are mean item score; all items were answered on a 4-point Likert scale (1–4); for all scales higher scores indicate higher levels of the construct.

Spearman rank correlations and point biserial correlations are given in Table 3. Positive associations were found between SumDental and age (e.g. older respondents reported more types of oral health problems), between psychophysical stress and dissatisfaction with trainer/team and between injury anxiety and unhealthy eating habits. Playing position showed a weak positive correlation with injury anxiety (e.g. slightly higher levels of injury anxiety were reported by external defenders, external and attacking midfielders and forwards).

**Table 3:** **Spearman rank and point-biserial correlations (n = 215)**

Variable	Injury anxiety^1^	Psyphy. stress^1^	Age^1^	Unh. eating h^1^	Dissatis-fact. t/t^1^	Playing position^2^
SumDental	.07	.13	.33^**^	.13	.13	.03
Injury anxiety		.11	-.04	.20^**^	.03	.15^*^
Psyphy. stress			.08	.06	.31^**^	.02
Age				.03	.15^*^	.02
Unh. eating h.					.00	.01
Dissatisfact. t/t						-.07

Legend: Psyphy. stress = Psychophysical stress; Unh. eating h. = Unhealthy eating habits, Dissatisfact. t/t = Dissatisfaction with trainer/team; ^1^: correlations in the column are Spearman correlations; ^2^: correlations in the column are point-biserial correlations; *:p < 0.05 (2-tailed); **p < 0.01 (2-tailed).

Table 4 present the crude odds ratio’s of the other covariates in relation to the reinjury variables. The odds of having a REAMC was higher for external defenders, external and attacking midfielders and forwards (OR 4.6, 95% CI 1.93-10.85) than for soccer players at other positions (reference category). Compared to soccer players with an injury anxiety score of 1–1.60 (first quartile, reference category), the odds of having a MTR were higher for soccer players with an injury anxiety score of 1.81 – 2.20 (third quartile, OR 3.41, 95% CI 1.58-7.34) and for players with scores above 2.20 (fourth quartile, OR 2.41, 95% CI 1.03-5.68). Similarly, higher odds of having a MR were found for soccer players with an injury anxiety score of 1.61 – 1.80 (OR 3.72, 95% CI 1.63-8.50), for players with scores of 1.81 – 2.20 (OR 3.67, 95% CI 1.67- 8.07) and for players with scores above 2.20 (OR 2.93, 95% CI 1.22-7.03) than those soccer players who reported an injury anxiety score of 1–1.6 (reference category). Compared to soccer players with a psychophysical stress score of 1–1.25 (first quartile, reference category), the odds of having a MR were higher for soccer players with a psychophysical stress score above 1.50 (fourth quartile, OR 3.45, 95% CI 1.47-8.09). There were no statistical significant associations observed between dissatisfaction with trainer/team, unhealthy eating habits, age categories and the reinjury variables.

**Table 4:** **Univariate logistic regression analysis of other covariates with reinjury variables (n = 215)**

	REAMC	MTR	MR
	OR (95% CI)	OR (95% CI)	OR (95% CI)
Injury anxiety			
1st Q (1.00 - 1.60)	reference category	reference category	reference category
2nd Q (1.61 - 1.80)	1.65 (0.61- 4.43)	1.94 (0.87- 4.36)	3.72**(1.63- 8.50)
3rd Q (1.81 - 2.20)	1.59 (0.62- 4.10)	3.41**(1.58-7.34)	3.67**(1.67- 8.07)
4th Q (2.21 - 3.40)	1.36 (0.46- 4.01)	2.41*(1.03- 5.68)	2.93*(1.22- 7.03)
Psychophysical stress			
1st Q (1.00 - 1.25)	reference category	reference category	reference category
2nd Q (1.26 - 1.42)	0.43 (0.16- 1.13)	0.49 (0.23- 1.03)	0.57 (0.27- 1.22)
3rd Q (1.43 - 1.50)	0.55 (0.22- 1.36)	0.74 (0.36- 1.52)	1.26 (0.62- 2.59)
4th Q (1.51 - 2.25)	0.99 (0.40- 2.46)	1.90 (0.84- 4.28)	3.45**(1.47- 8.09)
Dissatisfaction with t/t			
1st Q (1.00 - 1.17)	reference category	reference category	reference category
2nd Q (1.18 - 1.67)	0.87 (0.36- 2.10)	0.82 (0.39- 1.73)	1.17 (0.55- 2.49)
3rd Q (1.68 - 2.00)	0.43 (0.15- 1.29)	1.01 (0.45- 2.24)	1.22 (0.54- 2.76)
4th Q (2.01 - 3.83)	0.92 (0.35- 2.39)	0.82 (0.38- 1.84)	2.26 (0.99- 5.13)
Unhealthy eating habits			
1st Q (1.00 - 1.50)	reference category	reference category	reference category
2nd Q (1.51 - 2.00)	0.95 (0.34- 2.64)	1.59 (0.70- 3.59)	1.68 (0.75- 3.81)
3rd Q (2.01 - 2.50)	1.11 (0.36- 3.41)	1.73 (0.70- 4.27)	1.90 (0.77- 4.71)
4th Q (2.51 - 4.00)	1.49 (0.55- 4.03)	2.19 (0.96- 5.00)	2.19 (0.96- 5.00)
Age			
16-20 years	reference category	reference category	reference category
21-23 years	0.69 (0.27- 1.74	0.80 (0.39- 1.65)	0.71 (0.34- 1.47)
24-26 years	0.85 (0.29- 2.45)	0.50 (0.21- 1.20)	0.71 (0.30- 1.67)
27 years and older	1.16 (0.47- 2.84)	1.11 (0.52- 2.37)	1.19 (0.56- 2.54)
Playing position^a^	4.58***(1.93-10.85)	1.69 (0.97- 2.95)	1.31 (0.76- 2.27)

Legend: REAMC = Repeated Exercise-Associated Muscle Cramps; MTR = Muscle or Tendon Reinjury; MR = Multiple types of Reinjury; Dissatisfaction with t/t = Dissatisfaction with trainer/team; Q = quartile; *p < 0.05; **p < 0.01;. ***p < 0.001.

^a^reference qroup: lower intensity positions (goalkeeper, central defender and central defending midfielder).

The crude and adjusted odds ratios for SumDental in relation to the reinjury variables were similar (see Table 5). SumDental showed statistically significant associations with all reinjury variables when analyzed as a continuous variable (adjusted odds ratios above 1.5). Players with two or all three types of oral health problems had higher odds of having REAMC, of having MTR and of having MT (adjusted odds ranging from 2.48 to 3.40) when compared to players without any of the oral health problems (reference category).

**Table 5:** **Crude and adjusted odds ratios for SumDental in relation to reinjury variables (n = 215)**

Sum dental	REAMC		MTR		MR	
	Crude OR (95% CI)	Adj. OR^a^(95% CI)	Crude OR (95% CI)	Adj. OR^a^(95% CI)	Crude OR (95% CI)	Adj. OR^a^(95% CI)
As continuous variable	1.77* (1.12- 2.81)	1.82* (1.07- 3.12)	1.59* (1.10- 2.32)	1.57* (1.01- 2.45)	1.87** (1.28- 2.75)	1.88** (1.19- 2.97)
As ordered categorical variable						
0 = no oral health problems present	reference category	reference category	reference category	reference category	reference category	reference category
1 = one type of o.h.p. present	1.92 (0.84- 4.38)	2.39 (0.92- 6.22)	1.51 (0.82- 2.79)	1.56 (0.78- 3.13)	1.84 (0.99- 3.41)	2.16* (1.06- 4.39)
2 = two or more types of o.h.p. present	3.14* (1.24- 7.96)	3.33* (1.11- 9.96)	2.59* (1.20- 5.57)	2.48* (1.01- 6.09)	3.53** (1.61- 7.73)	3.40** (1.35- 8.59)

Legend: REAMC = Repeated Exercise-Associated Muscle Cramps, MTR = Muscle or Tendon Reinjury, MR = Multiple types of Reinjury; o.h.p. = oral health problems.

^a^adjusted by the covariates injury anxiety, psychophysical stress, unhealthy eating habits, dissatisfaction with trainer/team, age and player position, all these covariates were analyzed as categorical variables; *p < 0.05, **p < 0.01.

## Discussion

The results of this preliminary study indicate that poor oral health is associated with repeated exercise-associated muscle cramps, muscle or tendon reinjury and multiple types of reinjury. These associations persisted after adjustment for age, player position, injury anxiety, psychophysical stress, unhealthy eating habits and dissatisfaction with trainer/team.

Our findings using self-report to assess oral health are consistent with the study by Gay-Escoda et al. [7] which reported that two objective clinical indicators of oral health, the plaque index and the probing pocket depth, were associated with muscle injury in professional male soccer players. On the basis of these results it is tempting to suggest that poor oral health may belong to the class of intrinsic risk factors for sports injuries labeled ‘health problems’ by Bahr and Krosshaug [4]. Support for this suggestion comes from studies showing that (1) poor oral health is also associated with chronically higher levels of IL-6 and other cytokines [10-13], that (2) chronically higher levels of IL-6 and other cytokines are associated with fatique [12,13] and that (3) fatique is a serious risk factor for (re)injuries [14,15].

According to the neuromuscular theory of exercise-associated muscle cramps, muscular fatigue is not only a risk factor but is a requirement for cramping [14]. Therefore, our finding that REAMC was associated with player position and with SumDental, indicates that both physical effort and poor oral health are associated with the development of muscular fatigue.

Our finding that injury anxiety was associated with past muscle or tendon reinjuries and with multiple types of past reinjuries, but not with repeated muscle cramps, is consistent with findings of previous studies, indicating a higher level of perceived risk of injury after an injury [36,37]. In accordance with the stress-injury model of Williams and Andersen [38], we found that psychophysical stress was associated with multiple types of past reinjuries.

### Strength and limitations of this study

This study is unique in that it is the first to examine the associations between poor oral health and reinjury risk of male elite soccer player, after adjusting for other covariates related to injury variables, i.e. injury anxiety, psychophysical stress, playing position and unhealthy eating habits. However, there are some important limitations of this study that deserve consideration.

Firstly, this study used self-reports on gum problems, restorations and apex resections. Clinical assessment of gum problems offers a much more reliable estimation than self-reports, but many researchers have argued that self- reports of gum problems are accurate enough for epidemiological studies [39]. With regard to the self-reported restorations and apex resections, it may be noted that self-reports of these dental services have been found valid [28]. Because of the chronic nature of dental diseases due to the ongoing presence of risk factors, we think that the likelihood of caries or apical periodontitis is higher in players with restorations or apex resections.

Secondly, self-reports on reinjuries were used. The reliability of these reports may be restricted due to response bias (i.e. denial of vulnerability), careless response and lack of insight into the type of reinjury. However, as lower identification and reporting generally results in lower associations, the associations found in this study can be regarded as conservative.

Thirdly, the scales of the questionnaire used in this study, the Sports Injury Risk Indicator, were not validated in previous studies. However, the items of the scales were extracted from previously validated scales, including the Tampa Scale for Kinesiophobia, the Sport Anxiety Scale, the Langner Index and the Athlete Satisfaction Questionnaire. In this study we assessed whether the selected items contributed to the reliability of the scales in our sample; items that lowered Cronbach’s alpha were removed. In addition we presented the associations between the scales in the results section.

Fourthly, the respondents were not randomly selected from all professional soccer players. They were recruited from eight premier league clubs with a medical staff that had a positive attitude towards this functional approach. Moreover, because players from different clubs from only three countries were enrolled, generalization of the results may be further restricted.

Finally, as we used a retrospective cross-sectional design, the results offer only preliminary evidence for dental and psychosocial problems as intrinsic risk factors for reinjury. In addition, the generalization of our findings on reinjuries to injuries may be limited because of a more structural character of reinjuries compared with injuries. The relationships of sports injuries with dental and psychosocial problems may be somewhat weaker than the comparable relationships of sports reinjuries. Hence, the results of this study should be confirmed in a prospective study, in which self-reports of dental problems are substantiated by examination of orthopantomograms (OPTs), and injury and injury severity are recorded by teams’ medical staff. Also recommended is research into the effects of improving or treating dental and occlusal problems on (re)injury risk in the longer term.

## Conclusions

Our study found associations between poor oral health and the kinds of reinjury examined in this study, i.e. repeated exercise-associated muscle cramps, muscle or tendon reinjury and multiple types of reinjury. These associations persisted after adjusting for the other covariates, i.e. injury anxiety, psychophysical stress, unhealthy eating habits, dissatisfaction with trainer/team, age and player position. These results justify a more thorough examination of the impact of poor oral health on the reinjury risk of playing elite soccer.

Although this study is preliminary, the findings may stimulate the organisation of comprehensive dental and occlusal examination before the beginning of the soccer season, first as a contribution to the prevention of oral pathologies and a stimulation for oral hygiene, but also as a possible contribution to the prevention of reinjuries.

## Abbreviations

REAMC: Repeated Exercise-Associated Muscle Cramps
MTR: Muscle or Tendon Reinjury
MR: Multiple types of Reinjury
